# A novel Chr1-miR-200 driven whole transcriptome signature shapes tumor immune microenvironment and predicts relapse in early-stage lung adenocarcinoma

**DOI:** 10.1186/s12967-023-04086-7

**Published:** 2023-05-15

**Authors:** Simon Garinet, Audrey Didelot, Laetitia Marisa, Guillaume Beinse, Marine Sroussi, Françoise Le Pimpec-Barthes, Elizabeth Fabre, Laure Gibault, Pierre Laurent-Puig, Sophie Mouillet-Richard, Antoine Legras, Hélène Blons

**Affiliations:** 1 Assistance Publique-Hôpitaux de Paris, Department of Biochemistry, Pharmacogenetics and Molecular Oncology, European Georges Pompidou Hospital, Paris Cancer Institute CARPEM, 20 Rue Leblanc, 75015 Paris, France; 2 Centre de Recherche des Cordeliers, INSERM, Team Personalized Medicine, Pharmacogenomics and Therapeutic Optimization (MEPPOT), Université de Paris, Sorbonne Université, Paris, France; 3 Department of Genetics and Molecular Medicine, Georges Pompidou European Hospital, APHP Centre, Paris, France; 4 Department of Thoracic Surgery, Georges Pompidou European Hospital, APHP Centre, Paris, France; 5 Department of Thoracic Oncology, Georges Pompidou European Hospital, APHP Centre, Paris, France; 6 Department of Pathology, Georges Pompidou European Hospital, APHP Centre, Paris, France

**Keywords:** Resected lung adenocarcinoma, Prognostic biomarkers, miR-200 signature

## Abstract

**Background:**

In Lung adenocarcinoma (LUAD), targeted therapies and immunotherapies have moved from metastatic to early stage and stratification of the relapse risk becomes mandatory. Here we identified a miR-200 based RNA signature that delineates Epithelial-to-mesenchymal transition (EMT) heterogeneity and predicts survival beyond current classification systems.

**Methods:**

A miR-200 signature was identified using RNA sequencing. We scored the miR-200 signature by WISP (Weighted In Silico Pathology), used GSEA to identify pathway enrichments and MCP-counter to characterize immune cell infiltrates. We evaluate the clinical value of this signature in our series of LUAD and using TCGA and 7 published datasets.

**Results:**

We identified 3 clusters based on supervised classification: I is miR-200-sign-down and enriched in *TP53* mutations IIA and IIB are miR-200-sign-up: IIA is enriched in *EGFR* (p < 0.001), IIB is enriched in *KRAS* mutation (p < 0.001). WISP stratified patients into miR-200-sign-down (n = 65) and miR-200-sign-up (n = 42). Several biological processes were enriched in MiR-200-sign-down tumors, focal adhesion, actin cytoskeleton, cytokine/receptor interaction, TP53 signaling and cell cycle pathways. Fibroblast, immune cell infiltration and PDL1 expression were also significantly higher suggesting immune exhaustion. This signature stratified patients into high-vs low-risk groups, miR-200-sign-up had higher DFS, median not reached at 60 vs 41 months and within subpopulations with stage I, IA, IB, or II. Results were validated on TCGA data on 7 public datasets.

**Conclusion:**

This EMT and miR-200-related prognostic signature refines prognosis evaluation independently of tumor stage and paves the way towards assessing the predictive value of this LUAD clustering to optimize perioperative treatment.

**Supplementary Information:**

The online version contains supplementary material available at 10.1186/s12967-023-04086-7.

## Background

Lung cancer remains the major cause of cancer-related death in developed countries despite major advances in the management of metastatic disease. Non-small cell lung cancer (NSCLC) accounts for 80% of lung malignancies and, of these, lung adenocarcinomas (LUAD) is the predominant cancer type. Patients potentially curable by complete surgical resection have localized stage I-II-IIIA diseases and represent less than 40% of all lung cancer patients [1]. The treatment plan and relapse free survival of patients with LUAD are affected by many factors, but the TNM stage is the only parameter used to determine peri-operative treatments and evaluate prognosis [2]. Consensus guidelines support adjuvant treatment for patients with stage II and III based on trials showing that it associates with significant gain in survival of 5% at 5 years [3, 4]. However, all stage I to IIIA patients are at risk of relapse and death after surgery. Moreover, this strategy implies that many patients who would not have relapsed receive unnecessary chemotherapy. Thus, a better risk assessment is crucial to identify high-risk patients and optimize care. Recently, new peri-operative strategies have emerged. Adjuvant and neo-adjuvant targeted therapies and immunotherapies have been validated [5–8] or are under evaluation [9]. Improving risk stratification is warranted for many reasons, including the duration of therapy, the side-effects, the costs and the lack of clear demonstration of a gain in overall survival specially for patients with low relapse risk [5].

Recurrence after complete resection of NSCLC is related to micrometastatic cancer cells that may have acquired an invasive phenotype. Previous studies using public data sets have examined different pre-defined situations such as the expression of stem cell markers [10], the expression of hypoxia related markers [11] and of immune signatures [12] to evaluate prognosis. Others identified prognostic gene signatures without a priori hypothesis [13–16]. Tang et al. carried out a meta-analysis of 42 lung cancer prognostic signatures and reported that only half performed significantly better than random signatures for survival prediction. An increased expression of mesenchymal markers and a decreased expression of epithelial markers, referred to as epithelial-to-mesenchymal transition (EMT) is commonly associated to a gain of invasive properties. Although the exact role of EMT in tumour metastasis and cancer prognosis remains a matter of debate, there is a consensus on considering tumour plasticity as the major key point as it allows cells to switch back and forth from E to M states [17]. EMT is a complex molecular and cellular process of tissue remodelling that plays essential roles in cell invasion, migration and drug resistance in many cancer types including NSCLC [18]. Many signalling pathways control EMT including the transforming growth factor-β [19] and the epidermal growth factor (EGF) pathways, which activate transcription factors (TFs) [20], including SNAIL, ZEB [21, 22], and TWIST family members. Up-regulation of these TFs and loss of E-cadherin are hallmarks of EMT and are related to carcinogenesis and metastasis [23].

Because EMT is a complex molecular mechanism with dynamic changes, its scoring and evaluation within tumours remains challenging. The plasticity and reversibility of the EMT process further challenge the analysis of this phenotype. Tumours are often undergoing partial EMT and are in an hybrid state [24]. Transcriptomic-based signature and various scores have been developed to overcome this issue. Among those, the 76GS [25], KS [26] show good inter-correlations [27]. These signatures are considered as a satisfying estimation of the EMT status. However, their prognostic value is not clearly established. For example, a better OS is identified for patients with a KS high tumour, i.e. EMT low score in ovarian cancer (cohort mean HR [μ_HR_] = 0.68, *P* = 0.018), gastric cancer, (μ_HR_ = 0.7013), pancreatic cancer (μ_HR_ = 0.6006) and glioblastoma (μ_HR_ = 0.81). Concerning, breast cancer (μ_HR_ = 1.48; P = 0.006) or malignant melanoma (μ_HR_ = 1.48) the inverse relation is observed. For lung cancer, no correlation with OS was observed [26]. The 76GS was developed for NSCLC, and predicted resistance to EGFR inhibitors, but had no prognostic value on a series of metastatic patients [25].

EMT is related to important modifications of the micro-environment. The immune system is a determining factor for cancer initiation and progression and an important hallmark of cancer. In lung adenocarcinoma, EMT has been associated with immune cell infiltration [28, 29]. In NSCLC, it has been inversely associated with T-cell infiltration [30], and positively associated with expression of different immune checkpoint molecules, including PD-L1, and seems to favour tumour immune escape. In metastatic patients with epithelial cancers, a combined inflammatory and EMT signature predicted response to immunotherapy [31].

We previously showed in a series of localized 176 NSCLC, that neither EMT markers nor the EMT score correlated with outcome. However, the expression of miRs, known as core regulators of EMT, were strongly associated with disease free survival (DFS) and overall survival (OS). We showed that miR-200a, b and 429, located on chromosome 1, were the only EMT-related markers with a prognostic value both in DFS and OS [32]. The miR-200 family encompasses 5 miRs in 2 clusters: miR-200a, b, 429 located on chromosome 1 (Chr1-miR-200), and miR-200c, 141 located on chromosome 12 (Chr12-miR-200). These miRs are important for maintaining the epithelial phenotype by directly targeting and repressing the expression of key EMT genes (*ZEB1* and *ZEB2*). A complex feedback regulation loop allows, in turn, ZEB1 to regulate miR-200s, creating a regulation hub that may either facilitate EMT or MET depending on a subtle equilibrium [33].

Here, we aimed to identify an EMT-related Chr1-miR-200 signature that could refine the prognostic value of EMT in LUAD using transcriptomic data. We uncovered EMT heterogeneity using a deconvolution method to decompose each sample as a combination of low and high Chr1-miR-200 signature components. The low to high gradient was shown to be linked to tumour molecular alterations, immune infiltrates and relapse free and overall survival independently of other predictors, including stage. We validated our results using TCGA and 8 other published data sets. This study adds new insight to the evaluation of EMT as a prognostic marker and shows that a deconvolution approach is a validated method for depicting EMT heterogeneity.

## Methods

### Patients

This study, conducted at the European Georges Pompidou hospital, was approved by the “CPP Ile de France 2” ethics committee (nos. 2012-08-09 and 2012-08-09 A1) and registered in clinical trial.gov (NCT03509779). Patients with NSCLC treated by surgery for curative intent signed informed consent for research and tumor tissues banking. A series of 107 primary lung adenocarcinoma were prospectively collected from October 2011 to December 2014. Samples were stored frozen (− 80 °C) at the Biological Resources center and Tumor Bank Platform (PRB-HEGP BB-0033-00063) before nucleic acid extraction. Baseline demographics and clinical variables were collected using the Epithor national database, and survival data were updated using patients’ medical records.

### DNA/RNA extraction

Tumors were cut prepared on a cryostat and reviewed by the pathologist before DNA and RNA extractions. Mean tumor cell content was 52% ± 25; all samples with < 20% were excluded. DNA and RNA were extracted using QIAamp DNA Mini Kit (Qiagen) and miRNeasy Mini Kit (Qiagen) extraction kits; DNAs and RNAs were quantified by Qubit Fluorometric Quantitation (Thermo Fisher Scientific) and stored frozen.

### DNA NGS analysis

Samples were characterized for molecular alterations by targeted next-generation sequencing (NGS) (Ion AmpliSeq™ Colon-Lung Cancer Research Panel v2, Life Technologies™, Carlsbad, CA) as previously described [32].

### 3′RNA-seq

PolyA-RNAseq libraries were prepared using the QuantSeq 3′mRNA-Seq Kit FWD for Illumina (Lexogen™) according to the manufacturer's instructions. Libraries were sequenced on a NovaSeq6000. Targeted coverage was 10 M reads by sample, and mean coverage obtained was 12.4 M reads by sample. Mean Phred Quality Score was 35.04 IC95% [34.97–35, 11]. Fastq RNA-seq files were analyzed using a standard bioinformatical pipeline, with adaptations related to polyA sequencing. Briefly, reads were mapped by STAR (v2.7.2a) [34]. Count files were normalized (edgeR Rpackage) to get log2-counts-per-million (logCPM) gene expression data [35, 36]. All genes with a HGNC symbol were kept.

### EMT score calculation

Various signatures to quantify EMT status in tumors have been published. We used 2 methods analyzed and compared by Chakraborty et al. [27], designated as the 76 genes signature (EMT-76GS) and the Kolmogorov SmiRnov test signature (EMT-KS). R (version 4.2.1) algorithms can be accessed through the following link: https://github.com/priyanka8993/EMT_score_calculation. We also used our EMT-7-genes-score (EMT-7G) previously published [32] that correlates to the 2 others.

### Unsupervised hierarchical clustering, supervised classification and WISP

The EdgeR (v3.36.0) R package was used for data normalization and analysis, ComplexHeatmap (v2.11.1) package was used for clustering and heatmap generation. WISP (v 2.1) package was used for deconvolution (https://github.com/cit-bioinfo/WISP). For detailed information and workflow, see Additional file 5: Supplemental Methods.

All analysis have been performed on a MacbookAir, macOS Monterey Version 12.2.1

## Result

### Patients and tumors

Clinical characteristics are shown in Table 1: the average age of patients was 64 years, most were stage I and II and had lobectomy as surgical procedure with extend lymph-node dissection. The median follow-up duration was 42 months. Less than a half of the patients had adjuvant chemotherapy (*n* = 38) or radiotherapy (*n* = 14) and 32 patients had relapsed or died at 3 years. The most frequent genetic alteration was *KRAS* mutation (42/107) followed by *TP53* (37/107) and *EGFR* (17/107) (Additional file 2: Table S1). Molecular alterations in *TP53*, *KRAS* or *EGFR* were not linked to age, stage, relapse, or death at 3 years. *EGFR* mutations were more frequent in women (*p* = 0.03) and in non-smokers (*p* = 0.006) and *TP53* mutations were more frequent in smokers (*p* = 0.03). No relation with tobacco exposure was identified in the *KRAS*-mutated group.

**Table 1:** Clinical features of patients

Clinics		
Sex ratio (F/M)		40/67
Age (y) ± SD		64 ± 11
Comorbidities		
Cardio-vascular		45 (42%)
Diabetes		15(14%)
Surgery		
Surgical resection		
Sub-lobar n (%)		9 (8%)
Lobectomy n (%)		89 (83%)
Bilobectomy n (%)		3 (3%)
Pneumonectomy n (%)		6 (5%)
Complete resection (R0) n (%)		107 (100%)
Side (D/G)		103/73
Oncology		
Histological type		
Adenocarcinomas n (%)		107 (100%)
TNM (IASLC 2009)		
Tx n (%)		3 (2%)
T1 n (%)		34 (32%)
T2 n (%)		49 (46%)
T3 n (%)		17 (16%)
T4 n (%)		4 (4%)
Nx		5 (5%)
N0 n (%)		62 (58%)
N1 n (%)		14 (13%)
N2 n (%)		26 (24%)
M0 n (%)		104 (97%)
M1 n (%)		3 (3%)
Stage		
I A n (%)		28 (26%)
I B n (%)		24 (22%)
II A n (%)		6 (6%)
II B n (%)		18 (17%)
III A n (%)		24 (22%)
III B n (%)		3 (3%)
IV n (%)		4 (4%)
Peri-operative treatment		
Neo-adjuvant chemotherapy		10 (9%)
Adjuvant chemotherapy		38 (36%)
Adjuvant radiation therapy		14 (13%)

### Non-supervised clustering

**Fig. 1:**
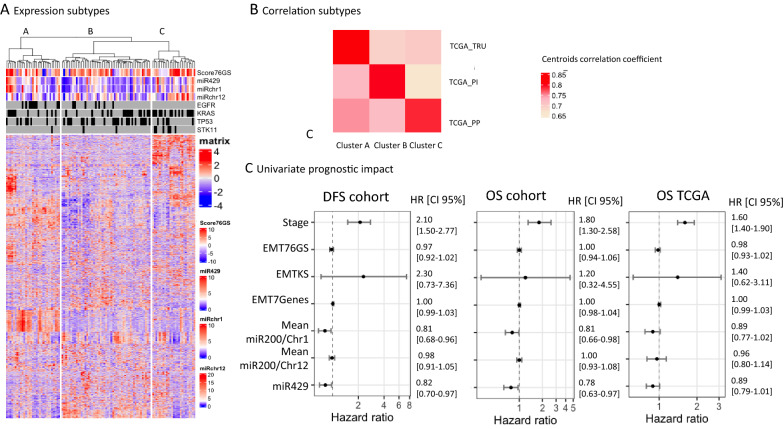
**A** Unsupervised analyses of 107 lung adenocarcinomas reveal significant interactions between molecular subtypes and mutation profiles. Tumors are displayed in columns grouped by mRNA expression. Genes are displayed and clustered in rows. Us and other have previously shown that the expression of miR-200s from chromosome 1 (miR-200s-chr1: miR-200a, b, 429) and from chromosome 12 (miR-200s-chr12: miR-200c, 141) were highly correlated within each chromosome group and pooled this information as a mean expression level for chr1 and chr12. **B** Centroids correlations of gene expression within each group reveals high concordance between the 3 clusters of the cohort, and 3 subtypes from TCGA (TCGA_TRU: Terminal respiratory unit, TCGA_PI: Proximal inflammatory, TCGA_PP: Proximal proliferative). **C** In univariate model, none of the EMT scores had correlation with outcome. Mean Mir-200/Chr1 expression or individual Mir-200/Chr1 (represented by MiR-429) expression are related to better outcome in DFS and OS both in the cohort, and TCGA data

We performed a non-supervised hierarchical clustering on RNA-seq data from our series of 107 resected lung adenocarcinoma. As published in the TCGA classification of lung adenocarcinoma [37] three distinct subtypes were identified (Fig. 1A). A centroid calculation based on the expression of the 1500 most variable genes in our series showed a high concordance between expression subtypes A, B and C of this dataset and the subtypes (TRU) “terminal respiratory unit”, (PI) “proximal inflammatory” and (PP) “proximal proliferative” defined in the TCGA dataset (Fig. 1B). Associated mutational profiles were similar between our clusters and TCGA. Subtype A was enriched in *EGFR* mutations (p = 0.003). Subtype B was enriched in *TP53* mutation (p = 0.03). Subtype C was enriched in *STK11* mutations (p = 0.001) and had no *EGFR* mutation.

The EMT score estimated by the 76 gene signature (see Methods) had a random distribution among the 3 groups (p = 0.43, Additional file 1: Fig. S1A). Conversely, the expression of miRs (mean Chr1-miR-200 or Chr12-miR-200 and miR-429) were significantly associated with tumor subtypes (p = 0.002, p = 0.001 and p = 0.024, respectively, Additional file 1: Fig. S1B–D). Tumors from subtype A had an intermediate expression of Chr1-miR-200 and low Chr12-miR-200, subtypes B had a low expression of all miRs and subtypes C had an intermediate expression of Chr1-miR-200 and the highest expression of Chr12-miR-200.

In our series as well as in the TCGA cohort, the EMT scores established by 2 published signatures or our EMT-7G signature showed no correlation with outcome neither in DFS nor in OS whereas miRs-chr1 (mean) or miRs-chr1 (individually; miR-429 showed as an example) did (Fig. 1C). Kaplan–Meier curves showed no impact of EMT score (76GS) in our cohort in DFS and OS (Additional file 1: Fig. S2A, B), nor on TCGA data (Additional file 1: Fig. S2C).

Based on the observation that (1) Chr1-miR-200 expression had a significant impact on non-supervised LUAD classification and (2) on prognosis, we performed a supervised classification based on miR-429 expression levels as a representative of Chr1-miR-200.

### Supervised classification and WISP

**Fig. 2:**
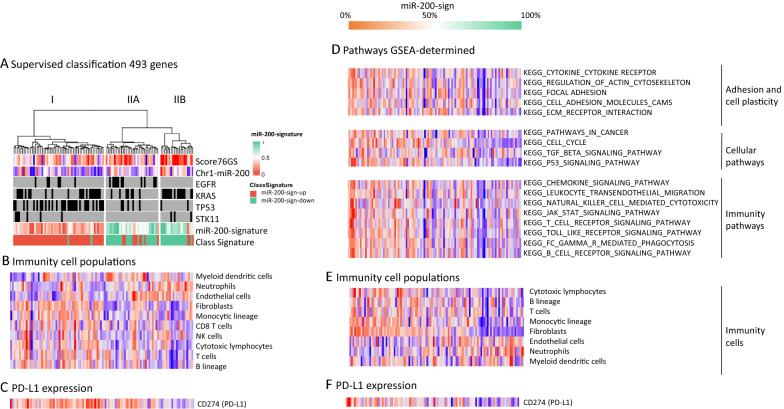
**A** Supervised classification of 107 lung adenocarcinomas based on the expression of miR-200 s reveals significant interaction between molecular subtypes and a high correlation with WISP classification. **B** Immune cell estimation determined by MCPcounter shows an enrichment in lymphocytes for tumors with miR-200-sign-down and an enrichment in myeloid cells for tumors with miR-200-sign-up. **C** PDL1 expression in clusters I IIA and IIB. **D** Gene set enrichment analysis (GSEA); tumors are ordered by impregnation of the miR-200-up-signature determined by WISP. Adhesion and cell plasticity, cell cycle, TGFbeta and TP53 and immunity are pathways enriched in tumors with a low proportion of the miR-200-up-signature, corresponding to “more mesenchymal”. **E** Immune cell estimation determined by MCPcounter shows an enrichment in lymphocytes for tumors with low miR-200-sign-up and an enrichment in myeloid cells for tumors with high miR-200-sign-up. **F** PD-L1 RNA expression

We used two approaches of supervised classification (Additional file 1: Fig. S3). First, tumors were ranked according to the expression of miR-429. The 15 tumors with the highest expression of miR-429 versus the 15 tumors with the lowest expression were tested for the 1500 most differentially expressed genes. Among these 1500 genes, 493 were common with the 1500 most variable genes from the non-supervised classification (Additional file 3: Table S2), and we performed a hierarchical clustering based on these 493 genes to visualize the influence of mir-200 related genes only on the unsupervised clustering (Fig. 2A). Secondly, we used weighted in silico pathology (WISP), to assess intra tumor miR-200-sign heterogeneity based on a centroid calculation of 150 genes (Additional file 4: Table S3). WISP results assigned 65 and 42 tumors in the miR-200-sign-down and miR-200-sign-up groups, respectively. Both supervised classifications were highly concordant with 87% (93/107) tumors identically classified. We identified three clusters: cluster I is miR-200-sign-down and enriched in *TP53* mutations (p < 0.001). Cluster IIA and IIB are miR-200-sign-up: cluster IIA is enriched in *EGFR* mutation (p < 0.001), cluster IIB is enriched in *KRAS* mutation (p < 0.001) and has a higher epithelial score based on 76GS (Fig. 2A). Interestingly, *EGFR* mutated tumors that cluster in groups I or IIA are associated to miR-200-sign-down or-up, respectively.

### Validation of miR-200 based clustering on TCGA data

The 493 genes set was then applied on LUAD from the TCGA dataset to validate the clusters, and WISP to score the proportion of miR-200 signature in each TCGA sample. WISP was trained on our cohort and applied to TCGA data (Additional file 1: Fig. S4A). Among TCGA samples, 197 were miR-200-sign-up and 242 were miR-200-sign-down. TCGA tumors are classified in 4 clusters and concordant with our data (Additional file 1: Fig. S4B). Cluster 1 from TCGA highly matches to cluster IIA/B in our cohort (miR-200-sign-up), and with IIA mutational profile. Cluster 4 matches to cluster IIB characterized by an absence of *EGFR* mutation. Finally, cluster 2 matches to cluster I (miR-200-sign-down) enriched in *TP53* mutations and cluster 3 highly matches with cluster I, and with less significance with cluster IIA.

### Gene enrichment in tumors with miR-200-sign-up and miR-200-sign-low

GSEA and MCP-counter were applied to identify set of genes enriched in the different subgroups and differences in immune cell infiltrates. Interestingly, pathways differentially enriched between miR-200-sign-low and -up samples were related to focal adhesion, actin cytoskeleton, and cytokine/receptor interaction, which is consistent with the implication of miR-200 in EMT, but also to TP53 signaling and cell cycle, which is concordant with the enrichment in *TP53* mutations in miR-200-sign-down tumors (Fig. 2D). Moreover, immunity and cytokine pathways were up-regulated in miR-200-sign-down tumors. Next, we evaluated the composition of the immune infiltrate with a deconvolution approach using the MCPcounter algorithm. As anticipated, the immune cell infiltrate highly differed according to miR-200-sign. MiR-200-sign-up tumors (epithelial subtype) showed enrichment in neutrophils, endothelial cells, low monocytes and a global lower infiltration in lymphocytes. As expected, fibroblast infiltration was inversely proportional to the miR-200-signature (Fig. 2E). MiR-200-sign-down tumors (mesenchymal subtype) were enriched in monocytes and lymphocytes but showed a higher PD-L1 mRNA expression, suggesting a possible exhaustion of the immune infiltrate (Fig. 2F). Supervised tumor classification yielded similar results (Fig. 2B, C). However, comparison of enrichment scores of immune cell infiltration between cluster IIA and IIB showed that the epithelial *KRAS* mutated subtype (IIB) is almost exclusively infiltrated by neutrophils (Fig. 2B) and, in agreement, shows lower expression of PD-L1 (Fig. 2C).

### miR-200-signature and survival

**Fig. 3:**
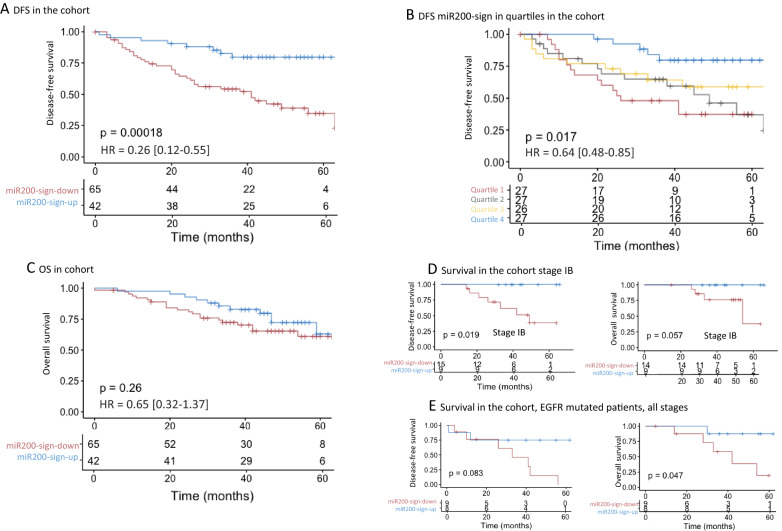
Survival relative to WISP miR-200-signature. DFS in the whole cohort (**A**) and in the cohort divided quartiles (**B**). OS in the whole cohort (**C**). Survival in stage IB tumors (**D**) and in *EGFR* mutated tumors (**E**), DFS and OS

The EMT score had no prognostic value in this cohort (Fig. 1C) nor in others (Additional file 1: Fig. S2). We had previously shown that Chr1-miR-200s were related to EMT and had a prognostic value [32]. We hypothesised that miR-200s had cellular effects beyond their direct impact on EMT targets and that the associated transcriptomic signature could also have a prognostic value. In our series, patients with miR-200-sign-up had a significant higher DFS than patients with miR-200-sign-down (median not reached after 60 months vs 41 months in Kaplan–Meier curves, p < 0.001) (Fig. 3A). When adjusted on stage, the strongest and validated prognostic marker, the association with a better DFS remained significant (p = 0.027). We showed that the miR-200-signature had a continuous effect on survival as, when analyzed in quartiles, an intermediate DFS was found for intermediate samples (Fig. 3B). Concerning OS, a trend for better survival was found in the miR-200 signature high group (Fig. 3C). Even though a higher proportion of miR-200-sign-up was found in stage I and II as compared to stage III, the prognostic value of the miR-200-sign remained high for stage I and II (Additional file 1: Fig. S5). To gain in clinical significance stage I were split into IA and IB. A strong prognostic impact was observed in stage IB with no relapse and death in the miR-200-sign-up group (Fig. 3D). Moreover, in the subgroup of *EGFR* mutated tumors, a trend for better prognostic value of miR-200-sign-up was also observed (Fig. 3E).

**Fig. 4:**
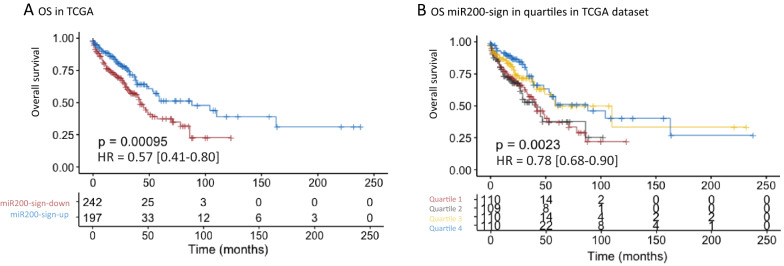
Overall survival relative to WISP miR-200-signature, (low proportion of miR-200-signature-up = mir200-sign-down; high proportion of miR-200-signature-up = mir200-sign-up) in TCGA data: (**A**) < 50% down or > 50% up, (**B**) divided in quartiles

The prognostic value of the miR-200-signature was confirmed on TCGA data. Patients with miR-200-sign-up had a significantly better OS than those with a miR-200-sign-down (median 42 months vs 59, p < 0.001) (Fig. 4A) and similar observations were obtained when the miR-200-signature was analyzed as quartiles (Fig. 4B). The “stage effect” identified in our cohort was confirmed on TCGA data, the prognostic value was maximum for stage I, and not significant for stage II and III (Additional file 1: Fig. S6).

Of note, our results were confirmed when applying the miR-200 signature to 7 available transcriptomic datasets (Additional file 1: Fig. S7), and the “stage effect” was also observed (Additional file 1: Fig. S8).

**Fig. 5:**
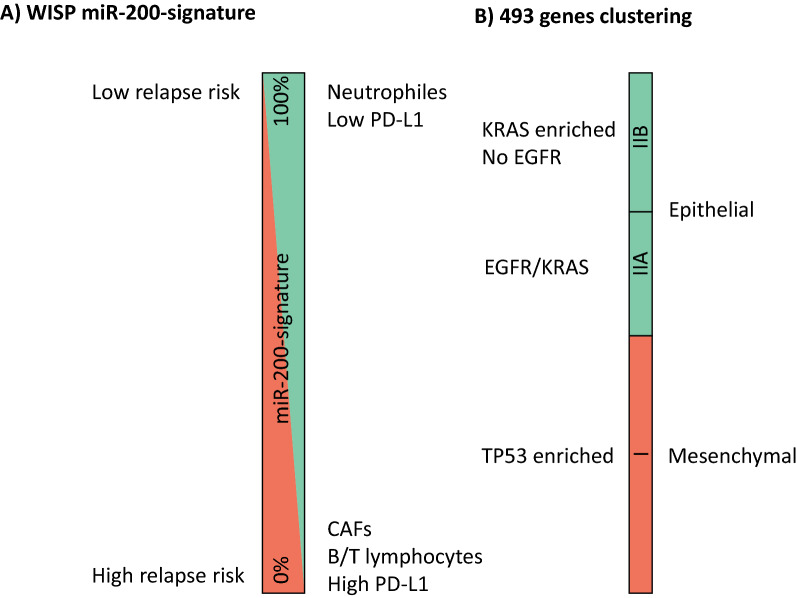
Graphical representation of main molecular and survival features driven by (**A**) the impregnation of the miR-200-signature determined by WISP and (**B**) the 493 genes signature supervised clustering

Main molecular features and prognostic implications are summarized in Fig. 5

## Discussion

Epithelial to mesenchymal transition affects tumor progression and metastasis but its characterization and its value, as a marker of prognosis remains debated. In NSCLC, low expression of E-cadherin, or high expression of EMT-transcription factors expression were not always related to better overall survival [38–42]. These discordant results highlighted the interest of considering EMT signatures. Transcriptomic-based signatures such as the 76GS [25] and KS [26] signatures and different scores based on canonical EMT markers have been developed to characterize score EMT [30] but in LUAD, most of them weakly associate with outcome. Recently, a novel EMT signature for prognosis prediction was published using public datasets and dbEMT 2.0 database [43]. In a previous work, we focused on EMT regulators and showed that decreased levels of miR-200a, b and miR-429 were significantly linked to DFS and OS in localized NSCLC [32]. Here we aimed to identify the associated transcriptomic signature. Non-supervised hierarchical clustering of LUAD shows 3 main clusters, in line with previous published data [37]. While EMT scores estimated by validated signatures had a random distribution among the 3 clusters, and a weak prognostic value, Chr1-miR-200 expression differed between groups, suggesting that miR-200 significantly impacted global gene expression. Based on this observation, we performed a miR-200 supervised clustering. Unsupervised and supervised classifications had 1/3 of the genes in common showing the strong impact of miR-200 on widespread gene expression. We assess intra-tumoral heterogeneity using WISP and quantified the impregnation of a miR-200-up signature as a continuous variable in each sample. The WISP-based classification was highly concordant with the supervised classification (Fig. 2A) and with the EMT 76GS signature (data not shown). An enrichment in signaling pathways relative to cell junction and plasticity, a higher infiltration of lymphocytes and a higher PD-L1 expression was noted in tumors with a low miR-200-up-signature impregnation. Pro-inflammatory tumor microenvironment and infiltrating CD8-expressing T lymphocytes are associated with improved survival in LUAD [44]. Here, tumors with a low proportion of miR-200 up signature, classified in cluster I (mesenchymal type), have higher lymphocyte infiltration as compared to others. However, this subgroup also has the highest proportion of monocytes and fibroblasts that have immunosuppressive functions and are PDL1 high, suggesting exhaustion of infiltrating lymphocytes. As EMT has previously been inversely correlated to T cell infiltration in NSCLC, it could be interesting to identify T cell subclasses and analyze their spatial organization. In ‘mesenchymal’ lung ADC, Chae et al. showed that tumors displayed a decreased infiltration of activated CD4 T-cells and a higher infiltration of activated B-cells but CD8 T-cells and regulatory T-cells (Tregs) were not significantly different [30]. Another recent publication showed an enrichment of macrophages, overexpression of checkpoint molecules, lymphocytes inhibitory cytokines, and immune exhaustion signatures in EMT-high tumors and especially in LUAD [45]. Concerning tumors in cluster II characterized by a higher proportion of the miR-200 up signature, IIA samples are significantly enriched in dendritic antigen presenting cells known to initiate and regulate immune responses only. IIB are low T and B cells, low dendritic cells and high neutrophils suggesting a degree of immune exclusion that could predict poor response to immune checkpoints inhibitors. Altogether, we show that the immune landscape varies according to the miR-200 signature. Whether miR-200s shape the immune tumor microenvironment or whether the microenvironment modulates miR-200 expression and induces tumor changes is still unclear at present. Here, the association between tumor clustering and molecular profiles suggests that molecular alterations themselves may drive EMT through miR-200 regulation. *TP53* is the most important feature associated with the miR-200 signature. Indeed, we found that *TP53* mutations are enriched in cluster I and in low miR-200-up signature. This observation has to be brought together with the reported positive regulation of the miR-200 family by wild-type *TP53* (reviewed in [46]). It is also in line with the high immune cell infiltration scores linked to *TP53* mutations[47, 48]. *TP53/KRAS* commutated samples also belong to this group, in agreement with the involvement of the miR-200/ZEB regulatory loop and the shift toward a mesenchymal phenotype demonstrated in *TP53*, *KRAS* double mutant cell lines [49]. We further showed that *EGFR* or *KRAS* mutated tumors split in different groups according to the proportion of the miR-200 signature. For instance, 1/3 of *EGFR* mutated tumors belong to the miR-200-down signature group. This may have implications regarding the response to osimertinib, which can now be considered as an adjuvant treatment for patients with *EGFR*-mutant NSCLC [5]. At present, the selection of patients eligible for treatment is based on stage. We argue that a better selection of high relapse risk patients using molecular markers could strengthen the benefit treatment. Indeed, we show that patients with *EGFR* mutated and miR-200 sign-down tumors are at higher risk of relapse independently of tumor stage, and, at the opposite, that patients with *EGFR* mutated and miR-200 sign-up tumors do not relapse and might not need adjuvant treatment. Moreover, the miR-200 signature delineated different immune profiles and association with response to immunotherapy should be analyzed. Finally, we saw that *KRAS* miR-200 sign-up tumors are characterized by low PDL1 expression, a poor immune infiltrate and a favorable prognosis as compared to *KRAS* miR-200 sign-down tumors. This subgroup might not benefit from perioperative immune checkpoint inhibitor treatments.

Another major finding of our study is that miR-200-sign-up tumors have a significant higher DFS than miR-200-sign-down. Of note, we show that the miR-200-signature was a very strong discriminant of relapse for early stages cancers in all tested cohorts. This is highly relevant in clinics when adjuvant treatment is debated. Regarding OS, the miR-200-signature had a weak association in our series. OS results may however be biased since we noted six non-cancer related deaths in patients without tumor recurrence in the miR-200-sign-up group versus none in the miR-200-sign-down group. OS may also be dependent upon the relapse profile such as local, second cancer or extra pulmonary metastasis but also on treatments, including second surgery or targeted therapies. Importantly, we validated the prognostic impact of the miR-200 signature on TCGA data, as well as on a large series of 7 transcriptomic datasets of lung adenocarcinoma publicly available.

The better stratification of patients is a major challenge in thoracic oncology. A clinical trial is ongoing to evaluate adjuvant chemotherapy in patients with intermediate or high stage I or stage IIA based on a 14 genes signature (NCT01817192). None of these 14 genes belongs our gene sets, suggesting a putative independent prognostic evaluation.

## Conclusion

The originality of our study was to use the miR-200 family, a main regulator of EMT, as a molecular basis for the definition a new transcriptomic signature, which we show to significantly contribute to LUAD classification, shape the tumor immune microenvironment and drive prognosis. Our work warrants our classification to be evaluated prospectively and to be considered as a useful tool for personalized management of early-stage LUAD patients.

## Supplementary Information

**Additional file 1: Figure S1.** Repartition of EMT Score 76GS (A), miR-429 expression (B), mean miR-200 s chr12 expression (C), mean miR-200 s chr1 expression (D). MiR expression is evaluated by ΔΔCt value relative to 3 reference miRs (y axis)[32]. **Figure S2** Kaplan–Meier curves of survival according to the EMT score estimated with the 76GS and divided in quartiles. In this cohort for DFS (A) and OS (B) and in TCGA data (C) for OS. **Figure S3** Schematic workflow of supervised classification. **Figure S4** Hierarchical clustering of 454 LUAD from the TCGA dataset based on the 493 genes reveals significant interactions between molecular subtypes (A) and a high correlation with WISP classification. The 4 clusters match the 3 clusters I, IIA/B identified in our cohort as demonstrated by a centroid correlation (B). **Figure S5** Disease-Free Survival relative to WISP miR-200-signature defined as < 50% of miR-200-signature-up = mir200-sign-down; > 50% of miR-200-signature-up = mir200-sign-up in our cohort adjusted by stage. **Figure S6** Overall Survival relative to WISP miR-200-signature defined as < 50% of miR-200-signature-up = mir200-sign-down; > 50% of miR-200-signature-up = mir200-sign-up in the TCGA LUAD cohort adjusted by stage. **Figure S7** Survival relative to WISP miR-200-signature defined as < 50% of miR-200-signature-up = mir200-sign-down; > 50% of miR-200-signature-up = mir200-sign-up among lung adenocarcinoma transcriptome available datasets. The p-values have been calculated by the log-rank test, HR = Hazard ratios estimated from a Cox univariate model, with the 95% confidence interval. Bild: https://doi.org/10.1038/nature04296. Chang: https://doi.org/10.1002/cncr.25592. Lee: https://doi.org/10.1158/1078-0432.CCR-07-4937. Roepman: https://doi.org/10.1158/1078-0432.CCR-08-1258. Shedden: https://doi.org/10.1038/nm.1790. Takeuchi: https://doi.org/10.1200/JCO.2005.03.8224. TomidaI: https://doi.org/10.1200/JCO.2008.19.7053. **Figure S8** Survival in early stages (I and II), compilation of the 7 lung adenocarcinoma available datasets.

**Additional file 2: Table S1**. Molecular alteration in the cohort.

**Additional file 3**: **Table S2:** 493 Common genes from unsupervised classification and supervised clustering based on miR-200 s (miR-429) expression.

**Additional file 4**: **Table S3:** 150 genes and centroids values determined by WISP.

**Additional file 5**: Supplemental methods.

## Data Availability

Materials, data, and protocols described in the manuscript will be made available upon reasonable request at the corresponding author.
