# Supplementary material for: A novel Chr1-miR-200 driven whole transcriptome signature shapes tumor immune microenvironment and predicts relapse in early-stage lung adenocarcinoma
Source: J Transl Med. 2023 May 15;21:324. doi: 10.1186/s12967-023-04086-7 (PMC10184345; doi:10.1186/s12967-023-04086-7)
Supplement: Supplementary file 5 — Additional file 5: Supplemental methods. [file 12967_2023_4086_MOESM5_ESM.docx]

**Supplemental Methods**

**EMT score calculation**

Various signatures to quantify EMT status in tumors have been published. We used 2 methods analyzed and compared by Chakraborty et al^28^, designated as the 76 genes signature (EMT-76GS) and the Kolmogorov SmiRnov test signature (EMT-KS). R algorithms can be accessed through the following link: <https://github.com/priyanka8993/EMT_score_calculation>. We also used our EMT-7-genes-score (EMT-7G) previously published^33^ that correlates to the 2 others.

**Unsupervised hierarchical clustering**

The EdgeR R package was used for data normalization and analysis. No normalization on expression was performed. The top 1500 most variable genes were selected, based on their standard deviation in the entire cohort. Classification was performed following the Ward’s method for columns and rows in ComplexHeatmap R package^38^. Distribution in the different clusters were performed by a Khi-square test for mutation status, and a Kruskal-Wallis test for expression levels.

**Supervised Classification and WISP**

A filter was applied on expression to keep genes with at least 10 counts in >10% tumors. MiR-429 was selected as the Chr1-miR-200 hallmark based on its high impact on prognosis^33^. However, miR-200a, miR-200b and miR-429 expression levels were highly correlated.

The 15 tumors with the highest and the 15 tumors with the lowest miR-429 expression levels were selected as comparison groups to identify a Chr1-miR-200 signature. Using edgeR package, following an exact test based on the qCML method we identified the 1500 genes the most differentially expressed. Classification was performed with ComplexHeatmap package in R v4.1.2.

WISP is a two-step approach that first estimates pure population profiles based on predefined pure samples and then estimates the proportion of these pure populations in mixed samples following the first step output. We used WISP to determine a signature based on the overexpression or the loss of expression of miR-429 and to quantify the proportion of the miR-up signature and the miR-down signature in each tumor. The 15 tumors with the highest and lowest expression levels of miR-429 were taken as input for WISP. Among these tumors, WISP selects the purest tumors and excludes the others. WISP kept 7 samples for the miR-200-down pure population, and 4 samples for the miR-200-up pure population (Supp. Fig. Met. 1A). To better classify tumors with a low tumor cellularity, a pool of normal lung tissues was added to create the pure normal entity and estimate the ratio of normal cells in tumors (Supp. Fig. Met. 1A and 1B). Tumors with the lowest cellularity estimated by the pathologist had the highest normal tissue estimation by WISP (Supp. Fig. Met. 1C) showing that WISP can provide a partial correction of expression data.

WISP identified 57 tumors as miR-200-down signature (miR-200-sign-down), 34 as miR-200-up signature (miR-200-sign-up), and 16 with a dominant normal tissue signature. We defined for each tumor a miR-200-up score as follows: proportion miR-200-sign-up / (proportion miR-200-sign-up + proportion miR-200-sign-down). This ratio is independent of the percentage of tumor cells in sample. Tumors with a ratio < 0.5 were classified as miR-200-sign-down, and tumors with a ratio >0.5 were classified as miR-200-sign-up. Altogether, 65 samples were miR-down and 42 were miR-up. The miR-200-up signature was used as an annotation to the heatmap of hierarchical clustering based on miR-200. Centroids used for pure entities are available in Supplemental Table 1. Among the 100 genes used by WISP for centroids calculations of miR-200-sign-up and miR-200-sign-down, 91/100 were common with the 1500 most differentially expressed genes. WISP R algorithm can be accessed through the following link: <https://github.com/cit-bioinfo/WISP>

Supplemental Figure of Methods 1


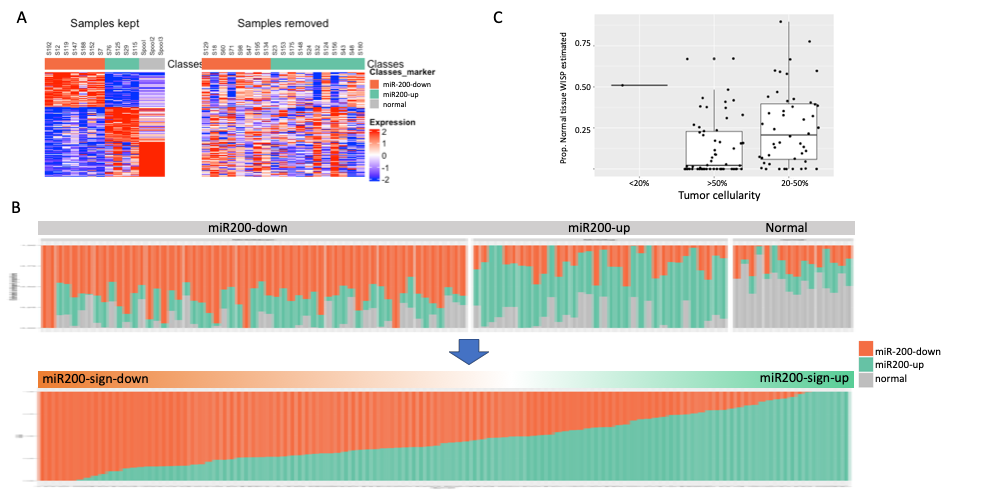


A) WISP algorithm kept the purest sample of each class to perform centroïd calculation for 150 genes and define the pure entities. B) WISP trained on pure entities is then applied to the cohort to estimate the proportion of each signature in individual tumor samples. C) Repartition of the proportion of normal tissue WISP estimated within each group of tumor cellularity defined in pathology
